# WTAP-mediated *N*^6^-methyladenosine modification of *NLRP3* mRNA in kidney injury of diabetic nephropathy

**DOI:** 10.1186/s11658-022-00350-8

**Published:** 2022-06-27

**Authors:** Jianzi Lan, Bowen Xu, Xin Shi, Qi Pan, Qing Tao

**Affiliations:** Department of Traditional Chinese Medicine, Shanghai East Hospital, Tongji University School of Medicine, No. 150, Jimo Road, Pudong District, Shanghai, 200120 China

**Keywords:** WTAP, NLRP3, Pyroptosis, High glucose, *N*^6^-methyladenosine, Inflammation

## Abstract

**Background:**

Diabetic nephropathy (DN) is prevalent in patients with diabetes. *N*^6^-methyladenosine (m^6^A) methylation has been found to cause modification of nucleotide-binding oligomerization domain, leucine-rich repeat, and pyrin domain-containing (NLRP) 3, which is involved in cell pyroptosis and inflammation. *WTAP* is a key gene in modulating *NLRP3* m^6^A.

**Methods:**

In this study, WTAP was silenced or overexpressed in high glucose (HG)-treated HK-2 cells to determine its influence on pyroptosis, NLRP3 inflammasome-related proteins, and the release of pro-inflammatory cytokines. NLRP3 expression and m^6^A levels were assessed in the presence of WTAP shRNA (shWTAP). WTAP expression in HK-2 cells was examined with the introduction of C646, a histone acetyltransferase p300 inhibitor.

**Results:**

We found that WTAP expression was enhanced in patients with DN and in HG-treated HK-2 cells. Knockdown of WTAP attenuated HG-induced cell pyroptosis and NLRP3-related pro-inflammatory cytokines in both HK-2 cells and db/db mice, whereas WTAP overexpression promoted these cellular processes in HK-2 cells. WTAP mediated the m^6^A of *NLRP3* mRNA that was stabilized by insulin-like growth factor 2 mRNA binding protein 1. Histone acetyltransferase p300 regulated WTAP expression. *WTAP* mRNA levels were positively correlated with NLRP3 inflammasome components and pro-inflammatory cytokines.

**Conclusion:**

Taken together, WTAP promotes the m^6^A methylation of *NLRP3* mRNA to upregulate NLRP3 inflammasome activation, which further induces cell pyroptosis and inflammation.

**Supplementary Information:**

The online version contains supplementary material available at 10.1186/s11658-022-00350-8.

## Background

Diabetic nephropathy (DN) is one of the most severe prevalent diseases in the modern world with a high morbidity rate [1]. 20 to 30% of diabetic patients have DN in type 1 and type 2 [2] and has been considered the main cause of chronic kidney disease. Studies have shown that inflammation and cellular injury induced by pyroptosis are closely associated with the development of DN and other kidney diseases [3–5]. However, the key mechanisms of DN development and DN-related cell pyroptosis and inflammation have not been entirely clarified.

The nucleotide-binding oligomerization domain, leucine-rich repeat, and pyrin domain-containing (NLRP) 3 inflammasome, which comprises NLRP3, apoptosis-associated speck-like protein containing a C-terminal caspase recruitment domain (ASC), and pro-caspase-1, is a vital part of the immune system preventing various infections [6]. NLRP3-induced inflammation is strongly associated with the pathogenic development of severe inflammatory disorders, including Alzheimer’s disease [7], DN [8], cryopyrin-associated periodic syndromes [9], and gout [10], among others. The activation of the NLRP3 inflammasome promotes the formation of the active form of caspase-1 by cleaving pro-caspase-1 [11]. Active caspase-1 further cleaves gasdermin D (GSDMD) to form GSDMD-N, which contains the N-terminal domain that attaches to membrane lipids and forms pores, thus leading to pyroptotic cell death [12, 13]. As GSDMD-N is active in inducing cell permeability, lysis, and the release of pro-inflammatory cytokines, it acts as a key factor in inducing inflammation [14, 15]. Additionally, IL-1β and IL-18 precursors are subjected to active caspase-1 cleavage for maturation [11, 16]. The mature forms of the pro-inflammatory cytokines are subsequently released into the extracellular milieu to recruit adjacent inflammatory cells [17]. Furthermore, high glucose levels have been shown to induce the pyroptosis of kidney epithelial cells [18, 19].

*N*^6^-methyladenosine (m^6^A), the most abundant internal RNA modification, is actively involved in the critical regulation of pre-mRNA processing, miRNA processing, translation initiation, and mRNA decay [20, 21]. The function of m^6^A is achieved by the synergetic and sophisticated association of methyltransferases, demethylases, and effector proteins [21]. Wilms tumor 1-associated protein (WTAP) is a key component of the classical m^6^A methyltransferase, which is responsible for stabilizing methyltransferase-like (METTL) 3 and METTL14 [22]. A study by Yang et al. indicated that the mRNA expression levels of *METTL3*, *METTL14*, and *WTAP* were significantly higher in patients with type 2 diabetes [23], while WTAP expression was also increased in plasma specimens of patients with diabetic retinopathy and in high-glucose-induced retinal pigmented epithelium cells [24]. Insulin-like growth factor 2 mRNA-binding proteins (IGF2BPs), known m^6^A readers, was found to promote tumor initiation and metastasis by stabilizing the m^6^A-containing mRNAs [25]. Moreover, SNP rs4402960 in the *IGF2BP2* gene, which encodes insulin-like growth factor 2 mRNA-binding protein 2, is significantly associated with an increased risk of type 2 diabetes [26]. Substantial evidence has shown that the epigenetic modification of DNA or RNA is crucial to the development of DN [27–29]. Woroniecka et al. have reported the increased expression of WTAP, NLRP3, and caspase-1 in patients with DN [30], suggesting a close association among m^6^A modification, pyroptosis, and DN development.

In the current study, we identified the upregulation of WTAP in DN. WTAP promoted NLRP3 inflammasome-dependent pyroptosis and inflammation in both in vitro and in vivo models. Mechanistically, the WTAP-mediated m^6^A modification led to the epigenetic upregulation of *NLRP3*, the mRNA of which was bound by IGF2BP1 for stability. Further investigations revealed that knockdown of *WTAP* significantly inhibited pyroptosis and NLRP3, pro-caspase-1, active caspase-1, GSDMD, GSDMD-N, IL-1β, and IL-18 levels. Our study is the first to validate the role of WTAP in pyroptosis and inflammation in DN.

## Materials and methods

### Ethics statement and clinical sample collection

This study was approved by the ethics committee of the Shanghai East Hospital and was conducted as per the Declaration of Helsinki. All participating patients submitted their written informed consent prior to the investigation. Human kidney biopsy tissues of patients (*n* = 63) with DN were obtained from the Shanghai East Hospital between August 2017 and January 2018, and normal kidney tissues from nephrectomies performed for renal hamartoma (*n* = 10) served as the control. All diabetic donors had type 2 diabetes. Diabetes status was defined as hemoglobin A1c ≥ 6.5%. Inclusion criteria required that: (i) donors be between 17 and 80 years of age, (ii) diabetic status be confirmed as noted above, and (iii) the postmortem time be within 24 h from cross-clamping in the operating room. The only exception with respect to hemoglobin A1c testing was if a donor had been prescribed and/or using glycemic agent(s), including but not limited to metformin and insulin, in which case repeated hemoglobin A1c testing was not mandatory. Exclusion criteria included active infection; malignancy; severe glomerulosclerosis, as determined in frozen operating room biopsy material; history of renal replacement therapy (hemodialysis or peritoneal dialysis at any time); and any known genetic renal condition, such as polycystic kidney disease. Tissue was placed into RNALater and manually microdissected at 4 °C for glomerular and tubular compartment. Glomeruli that readily released from the capsule and corresponding tubuli compartment were collected and placed into cold RNAeasy lysis buffer solution (RNeasy Mini Kit; Qiagen, Shanghai, China; 74106). All demographic data and anthropometric parameters were obtained at the time of enrollment.

### Immunohistochemistry

Immunohistochemical (IHC) staining was performed to validate WTAP protein expression following the standard protocol using the anti-WTAP antibody (ab195380; Abcam; 1:100 dilution), followed by incubation of the secondary antibody (D-3004; Shanghai Long Island Biotech Co., Ltd.). IHC evaluation was done on the basis of the percentage of positively stained cells (graded on a scale of 0–4: 0, < 5%; 1, 5–25%; 2, 25–50%; 3, 50–75%; 4, > 75%) and the intensity of staining (graded on a scale of 0–3: 0, negative; 1, weak; 2, moderate; 3, strong), which ranged from 0 to 12. Two pathologists who were blinded to the patients’ clinical and pathological characteristics conducted the immunohistochemical evaluation. The 63 patients were subsequently divided into two groups: low expression (IHC score < 4) and high expression (IHC score ≥ 4).

### Cell culture

Human renal tubular epithelial HK-2 cells and mouse kidney epithelial cell line (TCMK-1) were obtained from the American Type Culture Collection (ATCC, Manassas, VA, USA) and cultured in Dulbecco’s modified Eagle medium (DMEM; Gibco, USA; 10564029) with 10% fetal bovine serum (Gibco; 16000044) and 100 U/mL penicillin and streptomycin (Solarbio, Beijing, China; P1400-100). HK-2 cells were treated with 5.5 mM normal glucose (NG) and 30 mM high glucose (HG) for 48 h. The NG group was additionally treated with 24.5 mM mannitol to establish the control osmolarity.

### Adenovirus production

The human WTAP shRNAs and mouse WTAP shRNAs used were synthesized by Sangon Biotech Co., Ltd. (Shanghai, China). The shRNA sequence was cloned into the pShuttle-H1 adenovirus plasmids (shWTAP). Otherwise, the human WTAP coding sequence was synthesized and cloned into pShuttle-CMV adenovirus shuttle plasmids (Agilent, Beijing, China; 240007). The blank pAdEasy-1 adenovirus skeleton plasmid (Addgene Headquarters, Watertown, MA, USA; #16400) and pShuttle-H1-shWTAP or pShuttle-CMV-WTAP were co-transfected into HEK 293T cells using Lipofectamine 2000 (Invitrogen Life Technologies, Carlsbad, CA, USA) to produce a high-titer adenovirus vector. After 48 h transfection, recombined adenovirus plasmids were collected and transduced into HK-2 or TCMK-1 cells. Cells that were transduced with pShuttle-H1-nonspecific scramble shRNA plasmid (shNC) or blank pShuttle-CMV plasmid (vector) were used as the negative control.

### Transfection

HK-2 cells were transfected at 60–75% confluence with siRNA targeting IGF2BP1, IGF2BP2, IGF2BP3, or NLRP3 using Lipofectamine 2000 (Invitrogen) as per the manufacturer’s protocol. Scramble siRNA (siNC) for IGF2BP1, IGF2BP2, IGF2BP3, or NLRP3 were utilized as negative controls. The interfering RNA sequences are listed in Additional file 1: Table S1.

### Cell Counting Kit-8

HK-2 cells (3 × 10^3^ cells per well) were cultured in 96-well plates and incubated at 37 °C overnight. After 0, 12, 24, and 48 h treatment, 10 μL of the Cell Counting Kit-8 (CCK-8; Signalway Antibody LLC, College Park, MD, USA; CP002) solution was added into each well and incubated for an extra 1 h. Cell viability was subsequently determined using a microplate reader (PERLONG MEDICAL, Beijing, China; DNM-9602) at OD_450 nm_.

### Detection of cell pyroptosis

The cells were stained using FLICA 660-YVAD-FMK (FLICA 660 in vitro Active Caspase-1 Detection Kit, ImmunoChemistry Technologies, Bloomington, MN, USA; #9122) according to manufacturer’s instructions and with propidium iodide (PI; Invitrogen, Waltham, MA, USA; P3566) to mark cells with membrane pores. Cells were stained with 60× FLICA 660 and incubated for 1 h at 37 °C and protected from light. After which, the cells were washed three times with 1× Cellular Wash Buffer and stained with 3 μM PI for 15 min at 25 °C. The percentage of pyroptotic cells was then determined using a CytoFLEX flow cytometer (Beckman Coulter Cytoflex S, Krefeld, Germany). The output images from the cell pyroptosis assay included four fields, of which the field with active caspase-1^+^PI^+^ represents pyroptotic cells.

### Lactate dehydrogenase release assay

Pyroptosis was also evaluated by assaying the lactate dehydrogenase (LDH) released into the supernatants. A lactate dehydrogenase assay kit (Nanjing Jiancheng Bioengineering Institute, China; A020-2) was used to detect LDH.

### Real-time qPCR

RNA samples from human kidney tissues and HK-2 cells were extracted using TRIzol (Invitrogen; 15596026), and cDNA was generated using the Toyobo reverse transcription reagent kit (Takara, Japan; RR047A). Real-time qPCR (RT-qPCR) was performed using an ABI 7500 fast machine (Applied Biosystems, Foster City, CA, USA) with the SYBR Premix EX Taq kit (Takara; RR420A). The primer pair sequences are listed in Additional file 1: Table S2. Gene expression was normalized to that of glyceraldehyde-3-phosphate dehydrogenase (*GAPDH*) in either tissues or HK-2 cells. Relative quantification was determined using the 2^−ΔΔCT^ method.

### Western blot

Total protein samples from mouse kidney tissues and HK-2 cells were prepared in radioimmunoprecipitation assay (RIPA) lysis buffer (JRDUN Biotechnology Co. Ltd, Shanghai, China; BYL40825). Sodium dodecyl sulfate polyacrylamide gel electrophoresis (SDS–PAGE) was conducted to isolate protein samples, after which, the proteins were electrotransferred onto nitrocellulose membranes followed by blocking with 5% skim milk for 2 h at room temperature. The blots were incubated with primary antibodies against WTAP (Cell Signaling Technology, MA, USA; #56501), NLRP3 (Abcam; ab263899), caspase-1 (Abcam; ab207802), GSDMD (Affinity; AF4012), IGF2BP1 (Abcam; ab82968), IGF2BP2 (Abcam; ab129071), IGF2BP3 (Abcam; ab177477), H3K27ac (Abcam; ab4729), and GAPDH (Cell Signaling Technology; #5174) at 4 °C overnight. Incubation with goat anti-rabbit IgG (Beyotime, Shanghai, China; A0208) was subsequently performed for 1 h at room temperature. The western blotting bands were determined using ImageJ software.

### ELISA

The release of IL-1β (KAC1211; BMS6002TEN), IL-18 (KHC0181; KMC0181), TNF-α (KHC3014C; BMS607-3TEN), and IL-6 (BMS213-2; KMC0062 all from Invitrogen) in HK-2 cell supernatant and mouse serum was analyzed using ELISA kits according to the manufacturer’s instructions. The OD_450 nm_ of each sample was measured using a microplate reader.

### m^6^A content analysis

TRIzol reagent was used to extract total RNA. Poly(A)^+^ RNA was purified using GenElute mRNA Miniprep Kit (Sigma, Louis, MO, USA; MRN10). m^6^A content was assayed using the m^6^A RNA Methylation Assay Kit (Abcam, ab185912). Briefly, 80 µL of binding solution and 200 ng of sample RNA were added into each designated well, then incubated at 37 °C for 90 min for RNA binding. Each well was washed three times with wash buffer. Fifty microliters of the diluted capture antibody was added to each well, then incubated at room temperature for 60 min. Each well was incubated with detection antibody and enhancer solution at room temperature for 30 min subsequently. Finally, the wells were incubated with developer solution in the dark for 1–10 min at 25 °C. Reaction was stopped with stop solution and determined using a microplate reader at 450 nm wavelength within 2–10 min.

### RNA immunoprecipitation assays

RNA immunoprecipitation (RIP) assays were carried out using the Magna RIP RNA-Binding Protein Immunoprecipitation kit (Millipore, Billerica, MA, USA; 17-701) following the manufacturer’s protocol. The RNA–protein complexes were conjugated with anti-m^6^A (Abcam, ab208577), anti-IGF2BP1 (ab184305), or anti-IgG antibody (ab172730) at 4 °C for 1 h. Once incubation was complete, agarose beads and 50 µL of protein A/G were added and cells were incubated for a further 60 min at 4 °C. Subsequently, the precipitated beads were washed with RIP-wash buffer for 10 min at 4 °C and then RIP-lysis buffer for 5 min at 4 °C. The RNA in the immunoprecipitated complex and the RNA in the previously saved input fraction were released by incubating cells at 65 °C for 2 h with 200 mM NaCl and 20 µg proteinase K, which reversed the cross-linking. The coprecipitated RNAs were purified using phenol:chloroform:isoamyl alcohol and subjected to RT-qPCR.

### mRNA stability measurements

HK-2 cells were treated with 0.2 mM actinomycin D (GlpBio, Montclair, CA, USA; GC16866) for 30 min and were designated as the 0 h samples. The 2, 4, and 6 h samples were then collected for the extraction of total mRNA. cDNA synthesis by reverse transcriptase was subsequently performed using an oligo(dT) primer. The quantitated mRNA levels were determined by RT-qPCR.

### Chromatin immunoprecipitation (ChIP)

ChIP analysis was performed as previously described [31]. Briefly, cells with 10 μM C646 treatment were cross-linked in 1% formaldehyde, and the DNA was sonicated into a size range of 200–1000 base pairs using a Bioruptor Sonicator (Diagenode) for five cycles of 3 s on/3s off. The extracts were precleared in BSA-blocked protein A/G beads and incubated with anti-H3K27ac (Abcam; ab4729) or control IgG (Santa Cruz Biotechnology; sc-2027) overnight at 4 °C. After being washed, the DNA was eluted and reverse-cross-linked overnight at 65 °C; then, it was purified and confirmed by RT-qPCR (*WTAP* primers sequences: F, 5′-TTTCCACTCCCACCAGGAAAG-3′ and R, 5′-TAAGACTGCCATCTGGACCG-3′).

### Reporter gene assays

The *NLRP3* 3′UTR or 5′UTR sequence was cloned into the pGl3 vector (Promega, Madison, WI, USA). HK-2 cells were treated with 30 mM HG and transduced with WTAP shRNA or overexpression vector [cotransfected with either the pGl3-NLRP3 3′UTR or 5′UTR luciferase reporter plasmid and the pRL-TK vector (Promega) expressing the Renilla luciferase] using Lipofectamine 2000 (Invitrogen). Otherwise, H3 wild-type or K27R-mutant HK-2 cells, generated as previously described [32], were transfected with the pRL-TK vector and the pGL3-basic plasmid containing *WTAP* promoter sequence in the presence of 10 μM C646, a histone acetyltransferase p300 inhibitor, following the Lipofectamine 2000 (Invitrogen) transfection protocol. A dual-luciferase assay was conducted on the basis of the manufacturer’s protocol. Firefly luciferase activity was normalized to Renilla luciferase activity.

### Animals

A total of 20 C57BL/KsJ diabetic mice (db/db) (male; 8 weeks old) were housed under a 12 h:12 h light–dark cycle (7:00 am, on; 7:00 pm, off) (relative humidity: 55–60%; temperature: 22 °C ± 1 °C) and fed ad libitum with standard chow and water before being sacrificed. Another six C57BL/KsJ nondiabetic mice (db/m) (male; 8 weeks old) were used as control. The db/db mice exhibited obesity, hyperinsulinemia, and hyperglycemia as previously reported [33] and were randomly allocated into two groups, namely db/db + shNC group (*n* = 10) and db/db + shWTAP group (*n* = 10), wherein either control shNC or shWTAP were injected via the tail vein. A total of 12 mice (*n* = 6 per group) were successfully modeled for adenovirus injection via tail vein, with a success rate of 60.0%. Four weeks after injection, serum and urine samples were collected and mice were anesthetized by inhalation with 3% isoflurane, sacrificed by cervical dislocation, and kidney tissues were collected for H&E and Masson’s Trichrome staining as described in a previous study [34]. The serum creatinine, serum blood urea nitrogen (BUN), and urine protein levels were analyzed using a creatinine assay kit (C011-1-1), urea assay kit (C013-2-1), and urine protein test kit (C035-2-1; all from Nanjing Jiancheng Bioengineering Institute) as per the manufacturer’s instructions, respectively. Primary tubular segments were obtained from the cortex of mouse kidneys in each group and the primary tubular epithelial cells were cultured in DMEM/F-12 (Invitrogen) medium as previously described [35]. All procedures were authorized by the Institutional Animal Care and Use Committee (IACUC) and the ethics committee of of Shanghai Rat@Mouse Biotech Co., Ltd., China.

### Data analysis

All data in this study were processed using GraphPad Prism 8.4.2 and expressed as mean ± standard deviation (SD). We also used the Shapiro–Wilk normality test to evaluate the normal distribution of the collected data. When data showed normal distribution, two-sided unpaired Student’s *t*-test and one-way analysis of variance Tukey’s post-hoc test were adopted. *P*-value < 0.05 was considered statistically significant.

## Results

### WTAP is highly expressed in patients with DN and in HG-induced HK-2 cells

**Fig. 1:**
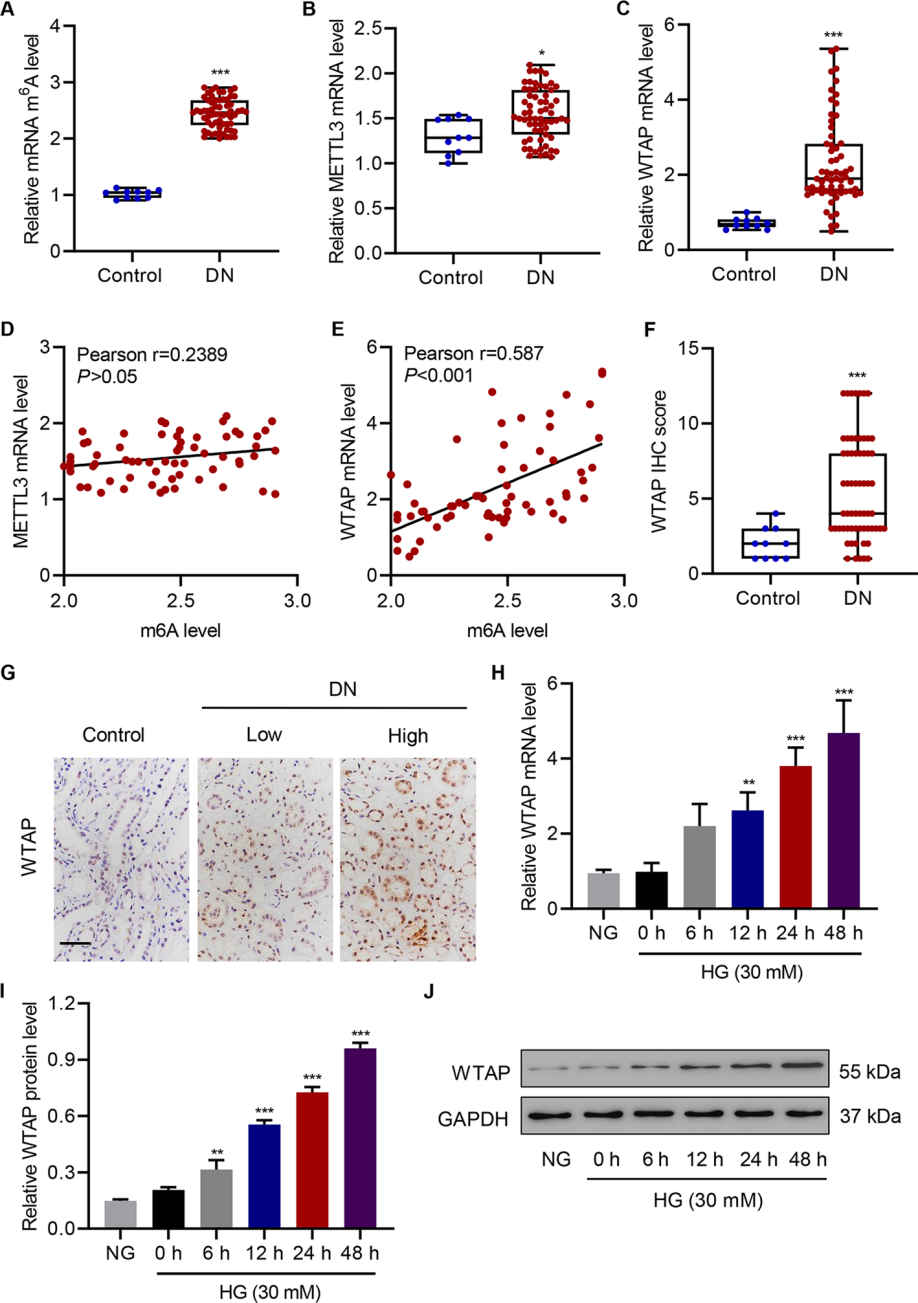
WTAP is highly expressed in DN tissues and HG-induced HK-2 cells. **A** m^6^A levels in control (*n* = 10) and DN tissues (*n* = 63) measured by ELISA. **B**, **C** mRNA expression of *METTL3* and *WTAP* in the control (*n* = 10) and DN tissues (*n* = 63) measured by real-time (RT)-qPCR. **D**, **E** Pearson correlation scatter plots (*n* = 63). **F**, **G** Protein expression of WTAP in the control (*n* = 10) and DN tissues (*n* = 63) measured by immunohistochemical staining. Scale bar, 50 μm. (**H**–**J**) mRNA and protein expression of WTAP in NG- or HG-induced HK-2 cells measured by RT-qPCR and western blot (*n* = 3). Unpaired Student’s *t*-test was used for the analysis between two groups, and one-way analysis of variance was used to analyze the data among multiple groups, followed by Tukey’s post hoc test. *P < 0.05, **P < 0.01, ***P < 0.001 compared with control or 0 h

To evaluate the role of WTAP in DN, we examined its expression in both DN tissues and HG-induced HK-2 cells. We first observed a significantly increased m^6^A level in patients with DN (Fig. 1A). Compared with the control group, DN tissues exhibited a 2.5-fold increase in m^6^A level, indicating high m^6^A modification activity. Interestingly, among the six m^6^A-regulating proteins, the *METTL3* and *WTAP* genes were highly expressed in renal tissues from patients with DN (Fig. 1B, C; Additional file 1: Fig. S1A–D). We further plotted the correlations between m^6^A level and *METTL3* or *WTAP* mRNA levels in renal tissues from patients with DN by calculating the individual Pearson’s coefficients (Fig. 1D–E). m^6^A modification levels were positively correlated with the mRNA levels of *WTAP* but not *METTL3*, suggesting that WTAP may be mainly responsible for m^6^A modification in DN. Since the DN showed not only disorder of renal tubules but also injury in glomerulus, m^6^A level and expression of WTAP were also detected in renal tubules and glomerulus, respectively. As shown in Additional file 1: Fig. S1E–H, m^6^A level and expression of *WTAP* were increased in renal tubules but not glomerulus from patients with DN. The IHC results similarly showed marked expression of WTAP in renal tubules from patients with DN (Fig. 1F–G). These data suggest that WTAP may play an important role in renal tubules in patients with DN. Moreover, WTAP expression was notably correlated with the clinical characteristics hemoglobin A1c (*P* = 0.023), hemoglobin (*P* = 0.038), eGFR (*P* < 0.001), BUN (*P* = 0.008), serum creatinine (*P* = 0.023), serum albumin (*P* = 0.011), and albuminuria (*P* = 0.027) (Additional file 1: Table S3). An in vitro model was also established using HK-2 cells (Fig. 1H–J). When subjected to HG treatment, WTAP expression was increased in a time-dependent manner, as indicated by RT-qPCR and western blot analysis.

### Knockdown of WTAP inhibits cell pyroptosis and pro-inflammatory cytokine release in HG-induced HK-2 cells

**Fig. 2:**
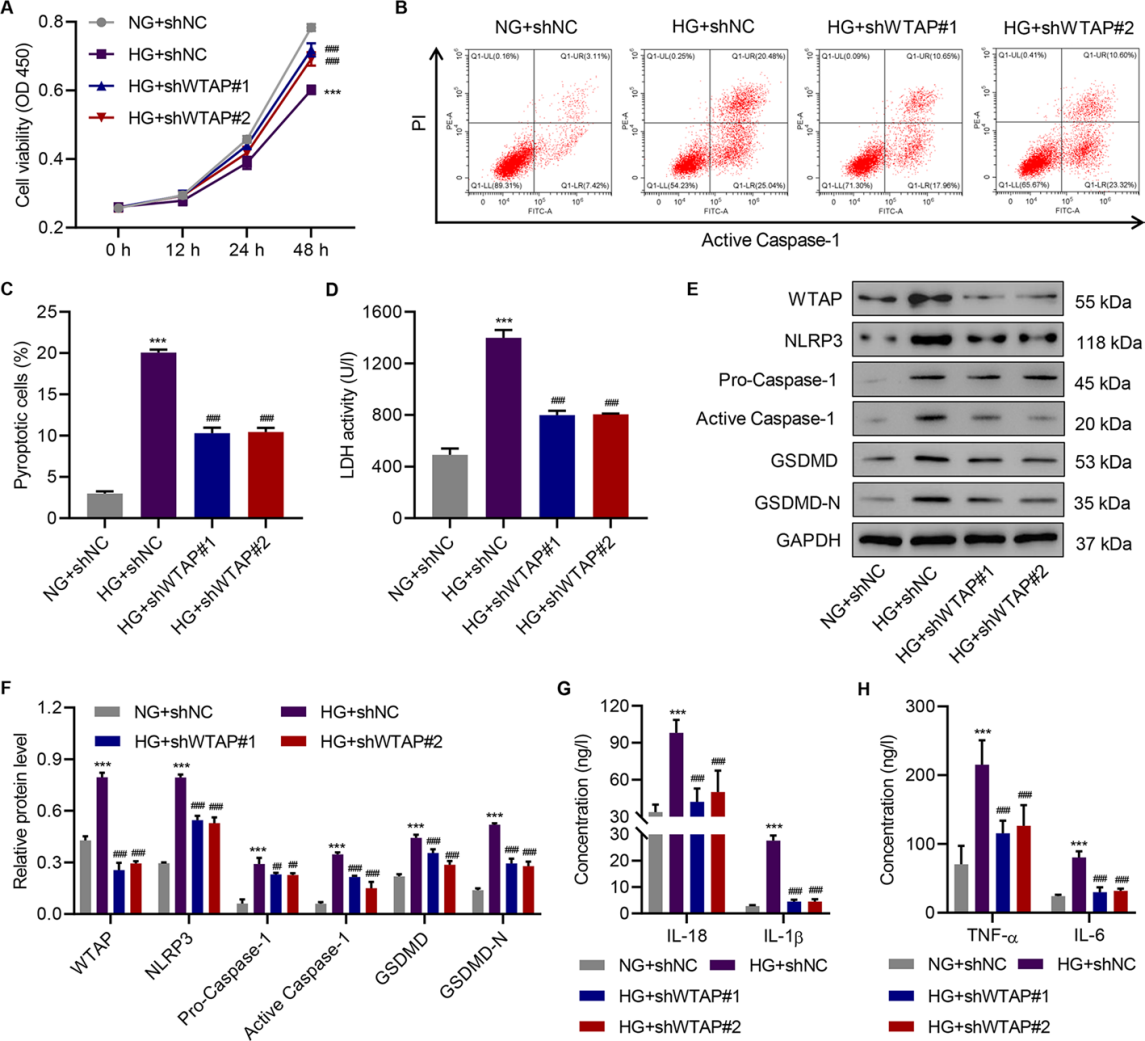
Knockdown of WTAP inhibits high glucose (HG)-induced cell pyroptosis and pro-inflammatory factor release in HK-2 cells. **A** Cell viability; (**B**, **C**) cell pyroptosis; (**D**) LDH activity; **E**, **F** expression of WTAP, NLRP3, pro-caspase-1, active caspase-1, GSDMD, and GSDMD-N; and (**G**, **H**) the release of IL-18, IL-1β, TNF-α, and IL-6 in HK-2 cells treated with NG or HG with or without WTAP knockdown (*n* = 3). One-way analysis of variance was used to analyze the data among multiple groups, followed by Tukey’s post hoc test. ***P < 0.001 compared with NG + shNC. ^##^*P* < 0.01, ^###^*P* < 0.001 compared with HG + shNC

To further investigate the function of WTAP in DN, we examined the effects of WTAP downregulation on HK-2 cells. Three shRNAs targeting WTAP were developed, which proved to effectively suppress its expression in HK-2 cells (Additional file 1: Fig. S2A–C). We initially determined the impact of shRNAs on cell viability. While monitoring the cell viability of HK-2 cells for 48 h, we observed reduced cell viability in the HG-treated HK-2 cells. However, shWTAP#1 and shWTAP#2 induced increased cell viability in the HG- and NG-treated HK-2 cells (Fig. 2A; Additional file 1: Fig. S2D). Flow cytometry also demonstrated that WTAP shRNAs inhibited cell pyroptosis in the HG-treated HK-2 cells (Fig. 2B, C). Likewise, the LDH release assay confirmed the potency of the shRNAs, suggesting that WTAP played a vital role in cell pyroptosis (Fig. 2D). Considering that the NLRP3 inflammasome is critical to cell pyroptosis induction, we first determined the changes in the protein levels of GSDMD and GSDMD-N (markers of pyroptosis) in HG-induced HK-2 cells after NLRP3 inflammasome inhibitor MCC950 treatment. As shown in Additional file 1: Fig. S2E, F, MCC950 significantly attenuated the increase in GSDMD and GSDMD-N induced by HG, which suggested that HG-induced pyroptosis was dependent on the NLRP3 inflammasome. Furthermore, we noted that HG stimulation also elevated the protein levels of NLRP3 and pro-caspase-1, which further elicited the incremental formation of active caspase-1. In comparison, the knockdown of WTAP attenuated the protein levels of NLRP3, pro-caspase-1, active caspase-1, GSDMD, and GSDMD-N (Fig. 2E, F). The release of downstream pro-inflammatory cytokines, including IL-18, IL-1β, TNF-α, and IL-6, in HG-treated HK-2 cells was detected in the presence of shWTAP#1 and shWTAP#2 (Fig. 2G, H). The release of all four cytokines was inhibited by the shRNAs. In short, HG treatment remarkably enhanced the release of the pro-inflammatory cytokines, whereas WTAP shRNAs significantly mitigated it.

### Overexpression of WTAP promotes cell pyroptosis and pro-inflammatory cytokine release in HK-2 cells

**Fig. 3:**
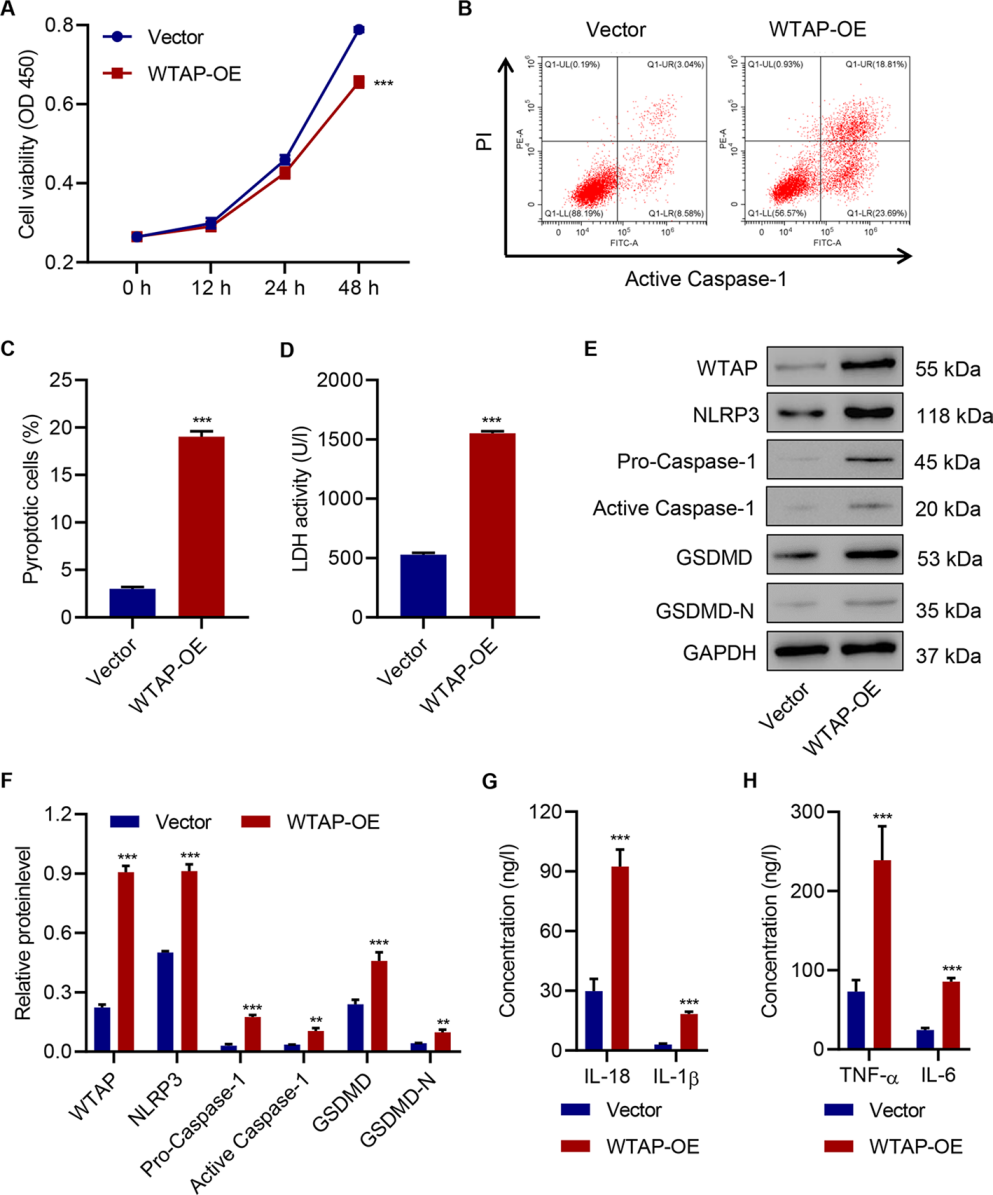
WTAP overexpression promotes cell pyroptosis and pro-inflammatory factor release in HK-2 cells. **A** Cell viability; (**B**, **C**) cell pyroptosis; (**D**) LDH activity; (**E**, **F**) expression of WTAP, NLRP3, pro-caspase-1, active caspase-1, GSDMD and GSDMD-N; and (**G**, **H**) the release of IL-18, IL-1β, TNF-α, and IL-6 in HK-2 cells with or without WTAP overexpression (*n* = 3). Unpaired Student’s *t*-test was used for the analysis between two groups. **P < 0.01, ***P < 0.001 compared with vector

To further evaluate the impact of WTAP on cell pyroptosis, we overexpressed WTAP in HK-2 cells (Additional file 1: Fig. S2A–C). We observed reduced cell viability in WTAP-overexpressing HK-2 cells in the absence of HG (Fig. 3A). Cell pyroptosis was largely promoted by WTAP overexpression (Fig. 3B, C), and LDH activity was increased (Fig. 3D). Moreover, the overexpression of WTAP influenced the levels of NLRP3 inflammasome components and active caspase-1 in HK-2 cells (Fig. 3E, F). Interestingly, GSDMD-N was barely produced in regular HK-2 cells in contrast to the significantly elevated levels after WTAP overexpression. Similarly, the release of pro-inflammatory factors was promoted by WTAP overexpression (Fig. 3G, H).

### WTAP targets NLRP3 in HG-induced HK-2 cells

**Fig. 4:**
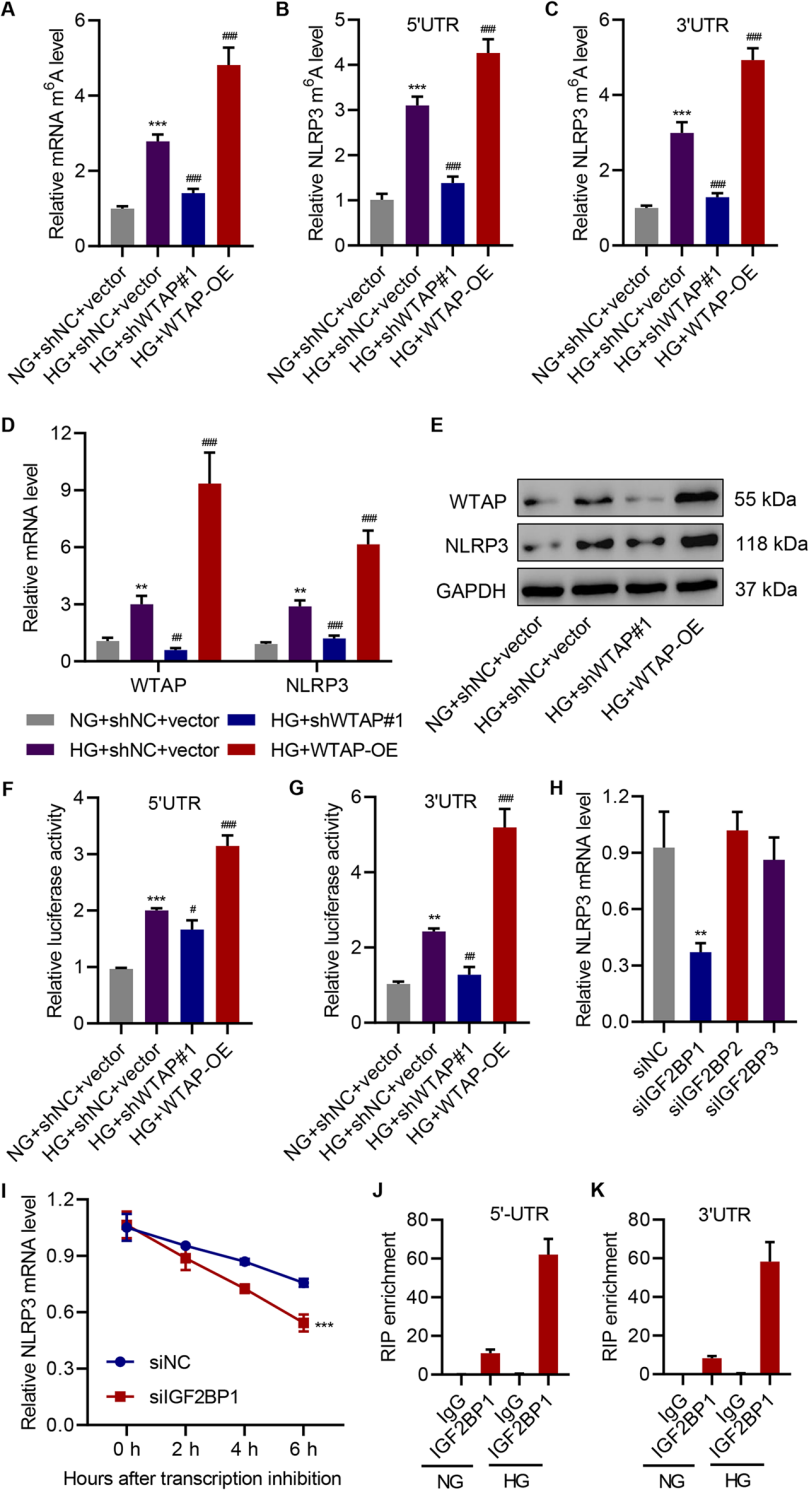
NLRP3 acts as a target of WTAP in high glucose (HG)-induced HK-2 cells. **A**–**G** HK-2 cells treated with NG or HG with or without WTAP knockdown or overexpression (*n* = 3). **A** m^6^A levels measured by ELISA; **B**, **C** RNA immunoprecipitation (RIP) using anti-m^6^A antibody and RT-qPCR analysis of *NLRP3* 5′UTR and 3′UTR m^6^A levels; **D**, **E** relative mRNA and protein expression of WTAP and NLRP3 detected by RT-qPCR and western blot; **F**, **G** luciferase activity of *NLRP3* 5′UTR and 3′UTR; **H** *NLRP3* mRNA level quantified by RT-qPCR in HK-2 cells with or without IGF2BP knockdown (*n* = 3); **I** half-life of the *NLRP3* transcript measured by RT-qPCR in HK-2 cells with or without IGF2BP1 knockdown (*n* = 3). **J**–**K** The binding of IGF2BP1 to *NLRP3* mRNA measured by RIP and RT-qPCR (*n* = 3). Unpaired Student’s *t*-test was used for the analysis between two groups, and one-way analysis of variance was used to analyze the data among multiple groups, followed by Tukey’s post hoc test. **P < 0.01, ***P < 0.001 compared with NG + shNC + vector or siNC. ^#^*P* < 0.05, ^##^*P* < 0.01, ^###^*P* < 0.001 compared with HG + shNC + vector

The previous experiments have suggested that WTAP induces cell pyroptosis through the NLRP3 inflammasome. We thus explored further the relationship between m^6^A modification and NLRP3 inflammasome-induced cell pyroptosis. Our experiments demonstrated that HG treatment resulted in a threefold increase of m^6^A levels in HK-2 cells, and WTAP overexpression synergistically contributed to a fivefold rise in m^6^A levels (Fig. 4A). In agreement with the previous results, knockdown of WTAP significantly attenuated the effects of HG treatment, thereby downregulating m^6^A levels. Some m^6^A modification sites of *NLRP3* mRNA such as 5′UTR and 3′UTR are predicted in RNAInter: RNA Interactome Database [36]. More importantly, both 5′UTR and 3′UTR of *NLRP3* underwent increased m^6^A modification when WTAP was overexpressed (Fig. 4B, C). We next confirmed the relationship between WTAP and NLRP3 levels (Fig. 4D, E). NLRP3 expression was positively correlated with HG treatment and WTAP expression. When WTAP expression was suppressed, NLRP3 expression was relatively low, and vice versa. The western blot results reinforced the regulatory role of WTAP in NLRP3 expression (Fig. 4E). Dual-luciferase assays indicated that the levels of both the 5′UTR and 3′UTR of *NLRP3* were affected by WTAP (Fig. 4F, G). IGF2BPs including IGF2BP1/2/3 regulate the enhancement of m^6^A-modified mRNAs stability. We performed the IGF2BPs depletion assay to examine further which IGF2BP was involved in NLRP3 expression (Additional file 1: Fig. S3A–C). We observed a decrease in *NLRP3* mRNA levels with the suppression of IGF2BP1 (Fig. 4H). The *NLRP3* mRNA stability was also decreased with the suppression of IGF2BP1 (Fig. 4I). RIP enrichment analysis showed that IGF2BP1 was able to bind to the 5′UTR and 3′UTR of the *NLRP3* mRNA, indicating that IGF2BP1 participated in the post-transcriptional regulation of *NLRP3* (Fig. 4J, K). Therefore, NLRP3 may serve as a regulatory target of WTAP-involved m^6^A modification in HG-induced HK-2 cells.

### WTAP promotes cell pyroptosis and inflammation in HK-2 cells by targeting NLRP3

**Fig. 5:**
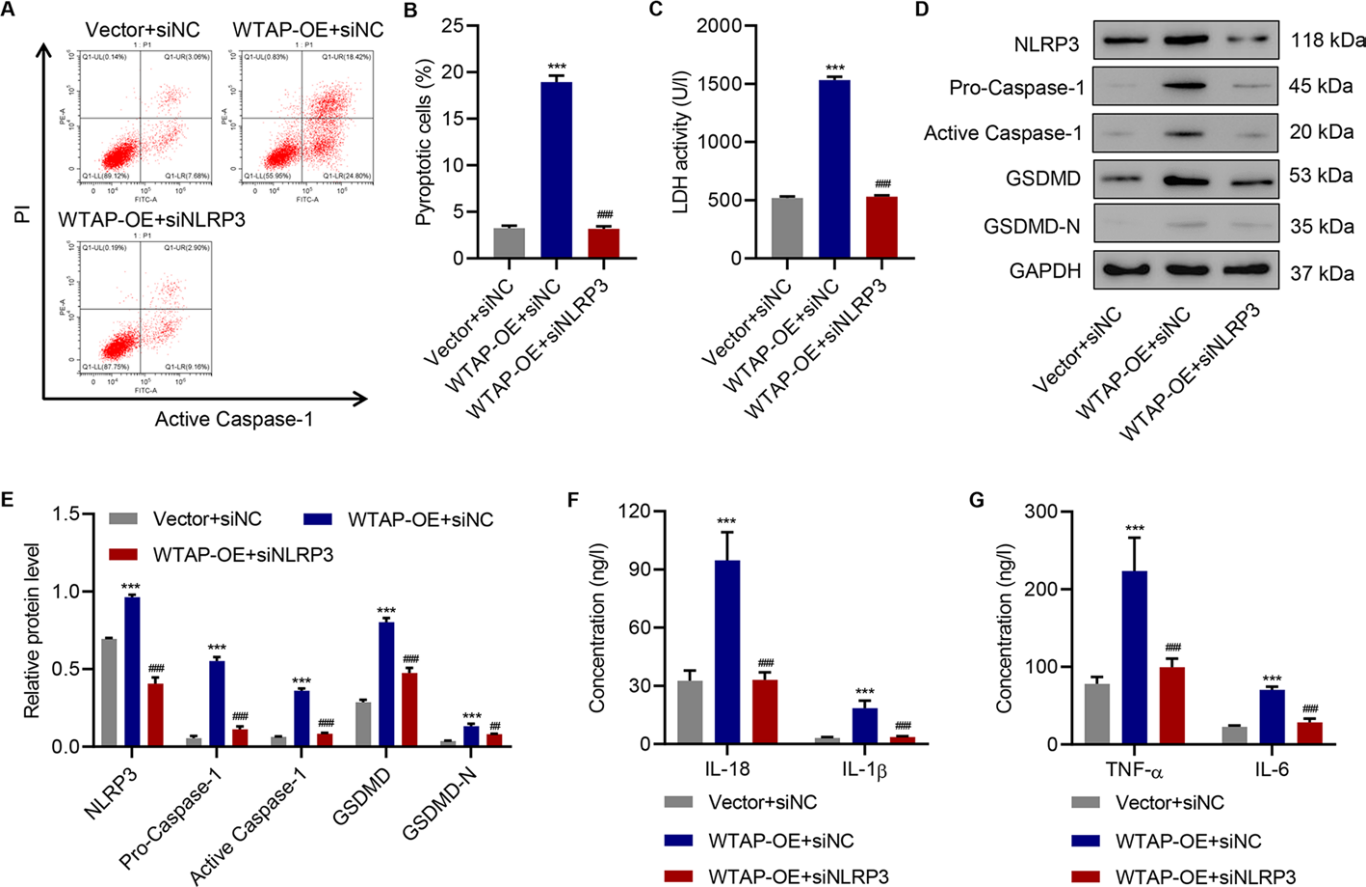
WTAP overexpression promotes cell pyroptosis and pro-inflammatory factor release in HK-2 cells by targeting NLRP3. **A**, **B** Cell pyroptosis; **C** lactate dehydrogenase activity; **D**, **E** expression of NLRP3, pro-caspase-1, active caspase-1, GSDMD, and GSDMD-N; (**F**, **G**) release of IL-18, IL-1β, TNF-α, and IL-6 in HK-2 cells with or without WTAP overexpression and NLRP3 knockdown (*n* = 3). ***P < 0.001 compared with vector + siNC. One-way analysis of variance was used to analyze the data among multiple groups, followed by Tukey’s post hoc test. ^##^*P* < 0.01, ^###^*P* < 0.001 compared with WTAP-OE + siNC

To further clarify the effects of NLRP3 in WTAP-induced cell pyroptosis and inflammation in HK-2 cells, NLRP3 was silenced (Additional file 1: Fig. S3D). We found that pyroptosis was significantly inhibited (Fig. 5A, B). Similarly, the LDH assay demonstrated that the knockdown of NLRP3 inhibited WTAP-induced increase of LDH activity (Fig. 5C). According to previous findings, WTAP overexpression facilitated the expression of NLRP3 inflammasome components. The knockdown of NLRP3 exerted inhibitory effects, not only on NLRP3 expression but also on the expression of other inflammasome-related proteins when WTAP was overexpressed (Fig. 5D, E). In addition, the release of major inflammatory factors was inhibited by the knockdown of NLRP3 (Fig. 5F, G).

### WTAP knockdown inhibits cell pyroptosis and pro-inflammatory factor release in db/db mice

**Fig. 6:**
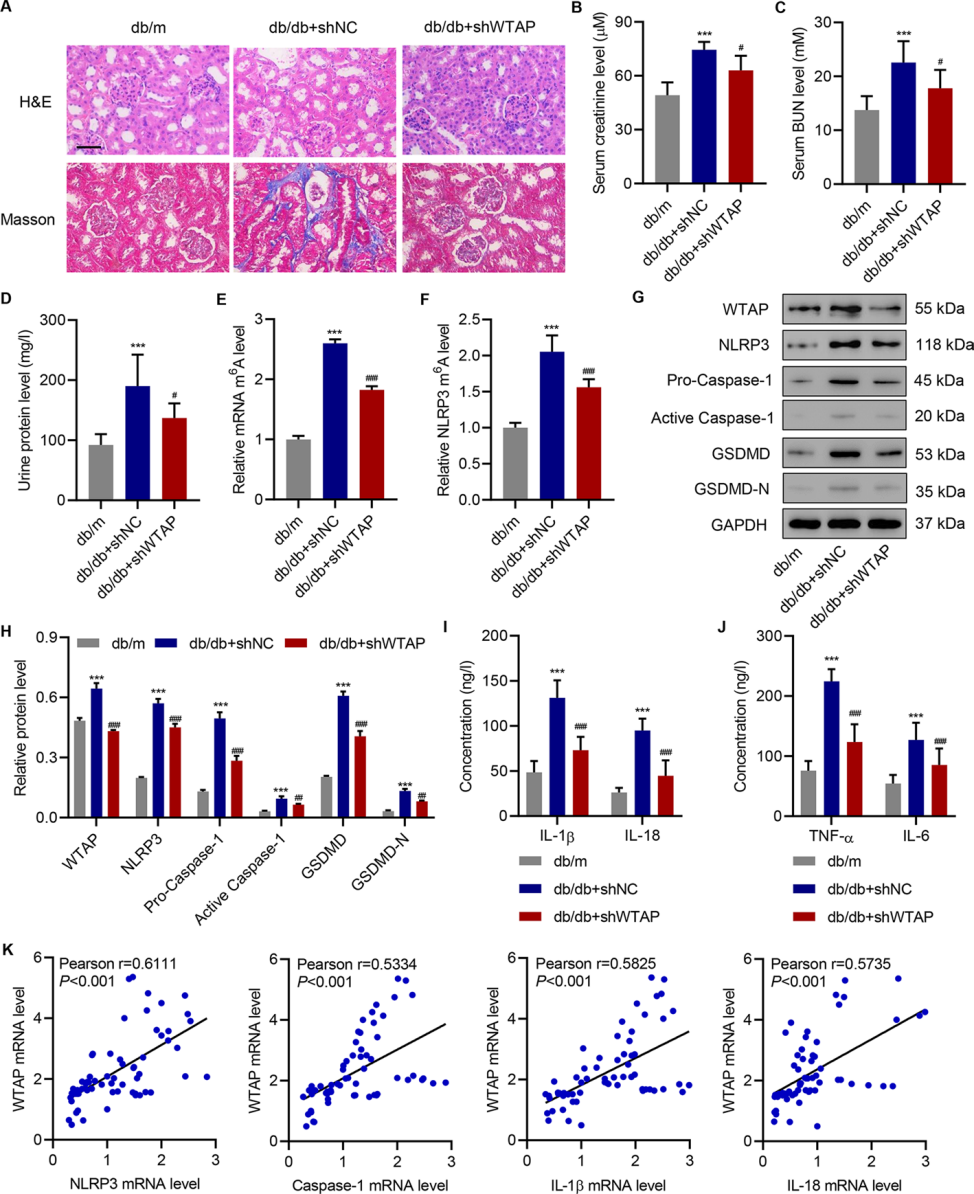
WTAP knockdown inhibits cell pyroptosis and pro-inflammatory factor release in db/db mice. **A** H&E and Masson’s trichrome staining of mouse kidney tissues (*n* = 6). Scale bar, 50 μm. **B**–**D** Serum creatinine, serum blood urea nitrogen, and urine protein levels (*n* = 6). (**E**) m^6^A levels measured by ELISA in primary tubular epithelial cells (*n* = 6). **F** RIP and RT-qPCR analysis of *NLRP3* m^6^A levels in primary tubular epithelial cells (*n* = 6). **G**, **H** Expression of WTAP, NLRP3, pro-caspase-1, active caspase-1, GSDMD, and GSDMD-N in primary tubular epithelial cells (*n* = 3). (**I**, **J**) Serum levels of IL-18, IL-1β, TNF-α, and IL-6 (*n* = 6). **K** Pearson correlation scatter plots (*n* = 63). One-way analysis of variance was used to analyze the data among multiple groups, followed by Tukey’s post hoc test. ***P < 0.001 compared with db/m + shNC. ^#^*P* < 0.05, ^##^*P* < 0.01, ^###^*P* < 0.001 compared with db/db + shNC

We developed shRNAs targeting mouse WTAP, which proved to effectively suppress its expression in TCMK cells (Additional file 1: Fig. S3E), and internalized shWTAP into the mouse. Renal tissue staining showed that the knockdown of WTAP notably inhibited renal damage and fibrosis in db/db mice (Fig. 6A). Compared with db/m mice, db/db mice maintained much higher levels of serum creatinine, serum BUN, and urine protein, indicating kidney damage and compromised renal functions. However, the use of shWTAP significantly reduced the levels of these markers (Fig. 6B–D). Similar to the findings in HK-2 cells, WTAP suppression enabled the downregulation of global and *NLRP3* mRNA m^6^A levels (Fig. 6E, F) and of NLRP3 inflammasome components in primary tubular epithelial cells (Fig. 6G, H). Furthermore, the serum levels of pro-inflammatory factors in db/db mice was largely inhibited by shWTAP (Fig. 6I, J). Therefore, the knockdown of WTAP can reduce cell pyroptosis in kidney tissue, restore kidney functions, and diminish pro-inflammatory factor release in db/db mice. To establish the relationship between WTAP expression and other critical proteins, we examined the changes in *WTAP* mRNA in kidney tissues of patients with DN. We quantified the *WTAP* mRNAs and other critical proteins and plotted the correlations by calculating the individual Pearson’s coefficients (Fig. 6K). *WTAP* expression was positively correlated with the mRNA levels of *NLRP3*, *caspase-1*, *IL-1β*, and *IL-18*. These data further supported the findings in HK-2 cell lines.

### Histone acetyltransferase p300 promotes WTAP transcription through H3K27 acetylation

**Fig. 7:**
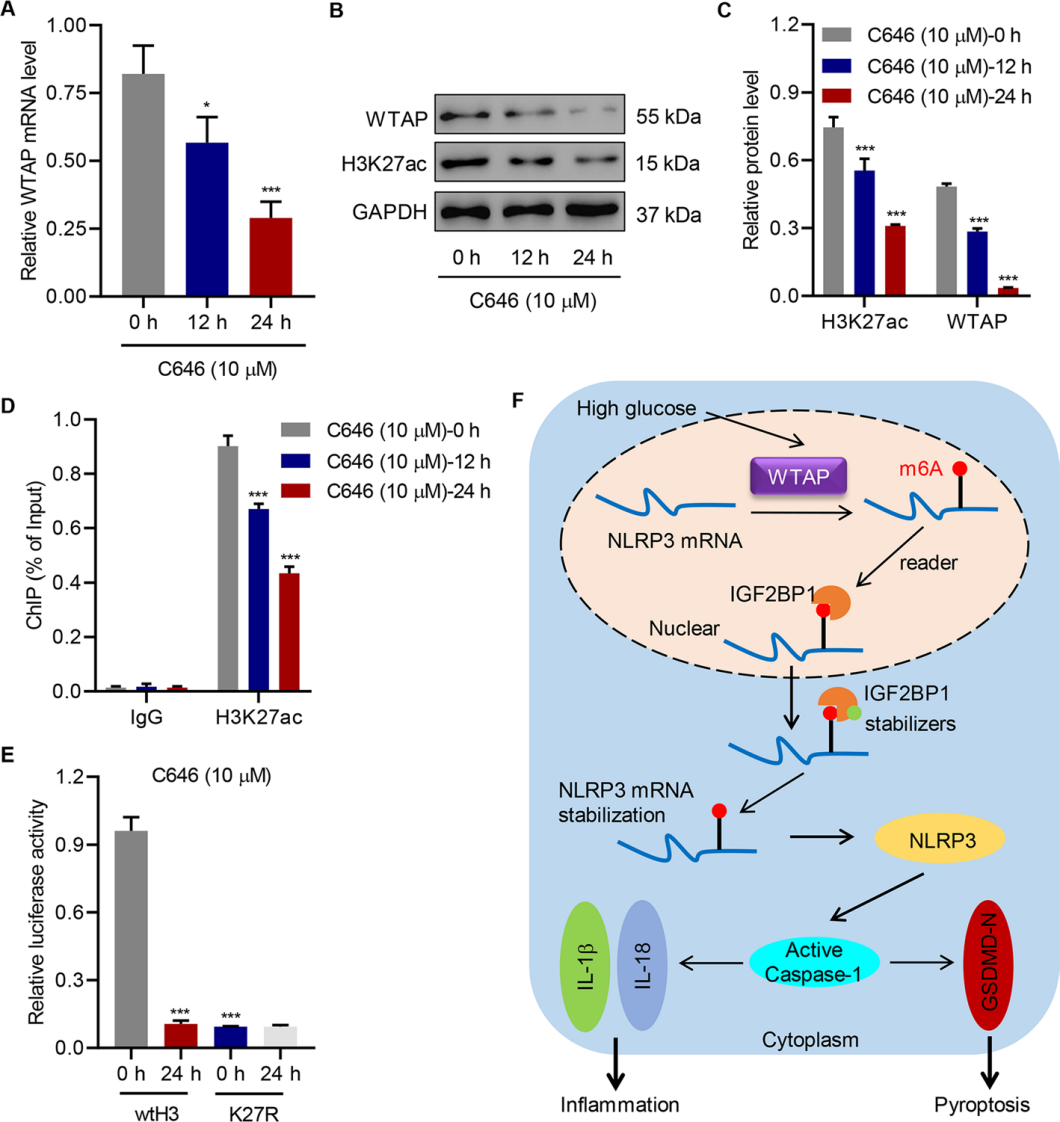
Histone acetyltransferase p300 promotes the transcription of the *WTAP* promoter through H3K27 acetylation. HG-induced HK-2 cells were treated with 10 μM histone acetyltransferase p300 inhibitor (C646). **A**–**C** WTAP expression and H3K27 acetylation (H3K27ac). **D** H3K27ac at *WTAP* promoter region. **E** Luciferase activity of *WTAP* promoter in H3 wild type (wtH3) or K27R-mutant (K27R) HK-2 cells treated with 10 μM C646 (*n* = 3). **F** Schematic diagram of the relationship between WTAP, m^6^A modification, HK-2 cell pyroptosis, and inflammation. One-way analysis of variance was used to analyze the data among multiple groups, followed by Tukey’s post hoc test. *P < 0.05, ***P < 0.001 compared with 0 h

Histone acetyltransferase p300 was reported to activate *METTL3* transcription in regulating m^6^A activity [37]. To prove that HG increases *WTAP* gene expression by affecting histones, we tested whether HG-induced WTAP expression could be inhibited by the C646, which reverses histone acetylation by inhibiting histone acetyl transferase p300 activity, in HK-2 cells. We found that the *WTAP* mRNA was significantly inhibited (Fig. 7A). The subsequent translation of WTAP was accordingly inhibited, along with H3K27ac expression (Fig. 7B, C). ChIP-RT-qPCR results showed that the binding between H3K27ac and *WTAP* promoter region was decreased in HG-induced HK-2 cells after being treated with C646 (Fig. 7D). HK-2 cells stably expressing H3-K27R protein were generated using the plasmid pIRESII-H3-K27R (referred to as K27R cells), encoding a bicistronic mRNA bisected by an internal ribosomal entry site. HK-2 cells transfected with pIRESII-H3 (referred to as wtH3 cells) were used as controls. We further engineered the *WTAP* promoter sequence into the dual-luciferase reporter vector for a luciferase assay. Downregulation of luciferase activity of *WTAP* promoter was observed in K27R cells as compared with the wtH3 cells, and no difference in luciferase activity of *WTAP* promoter in C646-treated K27R cells between 0 and 24 h was observed (Fig. 7E). These data indicate that p300 promoted *WTAP* transcription through H3K27 acetylation.

## Discussion

We described the correlation between WTAP and NLRP3 in pyroptosis and inflammation, which is crucial in the induction and development of DN. Evidenced by the active expression in both DN tissue specimens and HG-induced HK-2 cells, WTAP was positively correlated with m^6^A modification and promoted pyroptosis and inflammation of HK-2 cells by regulating the NLRP3 inflammasome. This upstream regulation occurred in the nucleus, in which *NLRP3* mRNA was m^6^A-modified by WTAP. The methylated *NLRP3* mRNA was stabilized by IGF2BP1. The resulting protein participated in the formation of the NLRP3 inflammasome, thus further regulating caspase-1-dependent inflammation and pyroptosis (Fig. 7F).

In a previous study, WTAP was found to be highly expressed in patients with DN [30]. However, the study fails to provide a clear clue about the regulation of WTAP expression. Histone acetylation may be a mechanism involved in the etiology of diabetes and the development of its complications. As an important histone acetyl transferase, p300 was shown to be upregulated in the retina and heart of diabetic animals [38]. Moreover, inhibition of p300 activity in monocytes grown in a HG environment led to a decrease in pro-inflammatory cytokines, suggesting a potential therapeutic target for DN [38]. Mosley et al. have presented the effects of HG on the regulation of insulin gene expression, in which histone acetyltransferase p300 was recruited to the promoter only at HG concentrations by interacting with the transcription factor Pdx-1 [39]. Moreover, p300-mediated H3K27ac modification, leading to NLRP3 inflammasome activation, potentially contributed to GSDMD-mediated pyroptosis [40]. Consistent with these observations, p300 promoted *WTAP* transcription through H3K27 acetylation. These findings collectively explain the HG-induced, p300-facilitated regulation of WTAP expression in HK-2 cells.

WTAP promotes renal cell carcinoma proliferation by regulating *CDK2* mRNA stability [41]. We have confirmed the function of WTAP in DN in promoting cell pyroptosis and inflammation in HK-2 cells and db/db mice. Moreover, WTAP silencing increased cell viability not only in HG-induced HK-2 cells but also in NG-treated HK-2 cells, which suggests that WTAP silencing increases cell proliferation independent of HG condition. Moreover, we have also established the connection between WTAP and the downstream effector protein, NLRP3. The NLRP3 inflammasome actively participates in a wide spectrum of diseases closely related to aberrant cell death [42–44]. The post-transcriptional regulation of *NLRP3* has been recently proposed to expand current understanding of NLRP3-dependent pyroptosis and inflammation [45]. We have confirmed the extensive m^6^A modification on *NLRP3* mRNA as regulated by WTAP in HK-2 cells. The knockdown of WTAP tremendously reduced m^6^A modification and further downregulated NLRP3 and pro-caspase-1 levels. Accordingly, the active form of caspase-1 was inhibited, thus resulting in a decrease of GSDMD-N. This finding explains the mechanism underlying WTAP-modulated pyroptosis and inflammation. Thus, the inhibition of WTAP is a potential therapeutic solution for DN. Pyroptosis occurs upon activation of pro-inflammatory caspases and their subsequent cleavage of GSDMD, resulting in GSDMD N-terminal fragments that form membrane pores to induce cell lysis [12, 13]. In the present study, pro-caspase-1, active caspase-1, GSDMD, and GSDMD-N expression was increased in high glucose- or WTAP overexpression-induced HK-2 cells and decreased by WTAP or NLRP3 silencing, respectively, indicating that cell death induced by WTAP overexpression and high glucose may be dependent on caspase-1 and GSDMD-mediated pyroptosis. Whether caspase-1 and GSDMD involved in high-glucose/WTAP-mediated pyroptosis should be further supplemented with data from application of caspase-1 and GSDMD inhibitor. Increased K^+^ efflux, intracellular reactive oxygen species (ROS) formation, and cathepsin B release are three major cellular events mediating NLRP3 inflammasome activation [46]. Previous studies have reported that ROS regulators such as GNAI3, TIGAR CYP1B1, and DDIT4 were the targets of WTAP in human cells [47–49]. Moreover, both WTAP and cathepsin B were upregulated in nonproliferative smooth muscle cells [50]. Therefore, further studies should be carried out to investigate the mechanism by which WTAP regulates activation of the NLRP3 inflammasome in DN. In addition to the release of inflammasome-dependent cytokines IL-1β and IL-18, inflammasome-independent cytokines TNF-α and IL-6 were also increased by WTAP or NLRP3. Meanwhile, IL-1β-induced NF-κB activation promoted the production of TNF-α and IL-6 [51]. These data suggest that the WTAP/NLRP3/IL-1β/NF-κB signaling axis may contribute to the induction of TNF-α and IL-6. Further studies will be required to identify a causal relationship between the above signaling axis and TNF-α/IL-6 in DN.

It has been shown that m^6^A methylation is a reversible process in some cases, and demethylases (erasers) play an essential role, such as FTO and ALKHB5 [21]. In the previous study, there were no differences in FTO and ALKHB5 expression in renal tubule between control and DN [30]. Therefore, the role of demethylases and whether the interference of demethylases can reverse the WTAP m^6^A methylation modification of *NLRP3* need to be further examined. Although the role of m^6^A modification and its regulators have attracted much attention, there are only a few papers published on their role in DN. Other studies reported upregulated METTL14 expression in renal biopsy samples from patients with DN and HG-induced glomerular endothelial cells and advanced glycation end product-induced podocytes, and METTL14-dependent m^6^A modification of Sirt1 and α-klotho mRNA contributes to podocyte and glomerular endothelial cell injury, respectively [52, 53]. However, the expression of WTAP was increased in DN renal tubules but not glomerulus compared with control. These data suggest that METTL14 and WTAP may play an important role in renal glomerulus and tubules, respectively. Moreover, the extensive m^6^A modification of *NLRP3* mRNA by METTL3 in HG-afflicted podocytes has also been confirmed [54]. METTL14 also increased the m^6^A modification of *NLRP3* mRNA in degenerative nucleus pulposus cells, while the reader decoding the m^6^A modification was different [55]. Variants in IGF2BP2 have been previously found to be significantly associated with alterations in insulin secretion and resistance, and IGF2BP2 was found upregulated in the β-cells of patients with type 2 diabetes [26]. In the present study, we observed that IGF2BP2 bound to *NLRP3* mRNA, which in turn stabilizes the *NLRP3* mRNA. It is not surprising that the m^6^A modification system could affect the progression of DN by many different mechanisms when considering the complexity of the modification system. Much more work needs be done to elucidate its role and mechanisms in cancer biology, which will inevitably provide new insights and, potentially, intervention strategies to fight against this deadly disease.

## Conclusions

Our current study reveals the key regulatory role of WTAP in promoting NLRP3-dependent pyroptosis and inflammation in human renal tubular epithelial cells. We present herein the first attempt to unveil the post-transcriptional m^6^A modification of . Furthermore, WTAP shRNA attenuated pyroptosis and inflammation in db/db mice, indicating a promising therapeutic solution that targets WTAP in DN.

## Supplementary Information

**Additional file 1: Table S1.** Interfering RNA sequences usedin this study. **Table S2.** Primer sequences used in this study. **Table S3.** Clinical characteristics of patients with DN and control subjects. **Fig. S1.** Expression of methyltransferases in DN tissues. **Fig. S2.** WTAP, GSDMD and GSDMD-N expression and cell viability in HK-2 cells. **Fig. S3.** IGF2BP and NLRP3 knockdown in HK-2 and TCMK-1 cells.

## Data Availability

The authors confirm the availability of all data generated or analyzed in this manuscript.

## Abbreviations

DN: Diabetic nephropathy
m^6^A: *N*^6^-methyladenosine
NLRP3: Nucleotide-binding oligomerization domain, leucine-rich repeat, and pyrin domain-containing 3
HG: High glucose
shWTAP: WTAP shRNA
GSDMD: Gasdermin D
WTAP: Wilms tumor 1-associated protein
METTL: Methyltransferase-like
IGF2BP1: Insulin-like growth factor 2 mRNA binding protein 1
IHC: Immunohistochemical
DMEM: Dulbecco’s modified Eagle medium
NG: Normal glucose
shNC: Scramble shRNA
siNC: Scramble siRNA
CCK-8: Cell Counting Kit-8
PI: Propidium iodide
LDH: Lactate dehydrogenase
RT-qPCR: Real-time qPCR
GAPDH: Glyceraldehyde-3-phosphate dehydrogenase
RIPA: Radioimmunoprecipitation assay
SDS-PAGE: Sodium dodecyl sulfate polyacrylamide gel electrophoresis
RIP: RNA immunoprecipitation
ChIP: Chromatin immunoprecipitation
BUN: Blood urea nitrogen
SD: Standard deviation
ANOVA: One-way analysis of variance
