# Supplementary material for: WTAP-mediated N6-methyladenosine modification of NLRP3 mRNA in kidney injury of diabetic nephropathy
Source: Cell Mol Biol Lett. 2022 Jun 27;27:51. doi: 10.1186/s11658-022-00350-8 (PMC9235192; doi:10.1186/s11658-022-00350-8)
Supplement: Supplementary file 1 — Additional file 1: Table S1. Interfering RNA sequences usedin this study. Table S2. Primer sequences used in this study. Table S3. Clinical characteristics of patients with DN and control subjects. Fig. S1. Expression of methyltransferases in DN tissues. Fig. S2. WTAP, GSDMD and GSDMD-N expression and cell viability in HK-2 cells. Fig. S3. IGF2BP and NLRP3 knockdown in HK-2 and TCMK-1 cells. [file 11658_2022_350_MOESM1_ESM.docx]

**Table S1.** Interfering RNA sequences used in this study

| Gene | Sequences (5′-3′) |
| --- | --- |
| Human WTAP shRNA#1  Human WTAP shRNA#2  Human WTAP shRNA#3  Human IGF2BP1 siRNA#1  Human IGF2BP1 siRNA#2  Human IGF2BP1 siRNA#3  Human IGF2BP2 siRNA#1  Human IGF2BP2 siRNA#2  Human IGF2BP2 siRNA#3  Human IGF2BP3 siRNA#1  Human IGF2BP3 siRNA#2  Human IGF2BP3 siRNA#3  Human NLRP3 siRNA#1  Human NLRP3 siRNA#2  Human NLRP3 siRNA#3  Mouse WTAP shRNA#1  Mouse WTAP shRNA#2  Mouse WTAP shRNA#3 | GCAAGTACACAGATCTTAA  GCGAAGTGTCGAATGCTTA  GGGCAACACAACCGAAGAT  GGACUUGGAGAAAGUGUUUTT  GGCUCAGUAUGGUACAGUATT  GAGCAAGAUACCGAGACAATT  CCCAGUUUGUUGGUGCCAUTT  GCGAAAGGAUGGUCAUCAUTT  GGAAGUGAUCGUCAGAAUUTT  CCUUGAAAGUAGCCUAUAUTT  GCUGCUGAGAAGUCGAUUATT  GGUGAAACUUGAAGCUCAUTT  GGAGAGACCUUUAUGAGAATT  CCACCAGAAUGGACCACAUTT  GCUUCAGGUGUUGGAAUUATT  GCACACCTGAGGATGACTT  GGAGGTAGTAGTTACATAA  GGTAATCGAACTGTGGGTT |

**Table S2.** Primes sequences used in this study

| Gene | Sequences (5′-3′) |
| --- | --- |
| WTAP-forward  WTAP-reverse  METTL3-forward  METTL3-reverse  METTL14-forward  METTL14-reverse  KIAA1429-forward  KIAA1429-reverse  RBM15-forward  RBM15-reverse  ZC3H13-forward  ZC3H13-reverse  IGF2BP1-forward  IGF2BP1-reverse  IGF2BP2-forward  IGF2BP2-reverse  IGF2BP3-forward  IGF2BP3-reverse  NLRP3-forward  NLRP3-reverse  IL-1β-forward  IL-1β-reverse  IL-18-forward  IL-18-reverse  Caspase-1-forward  Caspase-1-reverse  GAPDH-forward  GAPDH-reverse | ATGGCGAAGTGTCGAATGC  CCAACTGCTGGCGTGTCTC  TCTCCACGCCAGATGCTC  ACAGTCCCTGCTACCTCCC  CCTCCCATGTACTTACAAGCC  TAGCAGTGATGCCAGTTTCTC  GCAACTTCAGGCATTAAGTTCA  GTATTGCCTTGTCGAATCTGTC  GGCTGCCTGAGGAGAGTGGAG  CGGCTACTGCTCAATTCTGGACTG  GATCAGTTAAAGCGTGGAGAAC  CTCTCTGTCGTGTTCATATCGA  TCCCCGATGAGCAGATAGC  CTGGGTCTGTTTTGTGATGTTG  ATGAAACAGGGACCAAGATAAC  GTTGAAAAGATGCCAAGTGC  GATTAAATCTGAACGCCTTGG  TGGCACCGACTGATAGAGC  TTCGGAGATTGTGGTTGGG  GTCACCGAGGGCGTTGTC  ATCAGCCAGGACAGTCAG  GAAGCGGTTGCTCATCAG  GTTCAAGACCAGCCTGAC  GCTCACCACAACCTCTAC  GAAAGCCCACATAGAGAAG  CCCACAGACATTCATACAG  AATCCCATCACCATCTTC  AGGCTGTTGTCATACTTC |

**Table S3.** Clinical characteristics of patients with DN and control subjects

| Variables | Group | | | p-value |
| --- | --- | --- | --- | --- |
|  | Control (n=10) | DN with low WTAP level (n=28) | DN with high WTAP level (n=35) |  |
| Age (years) | 52.5 ± 10.2 | 49.6 ± 9.8 | 51.3 ± 10.7 | 0.800^a^; 0.672^b^ |
| Male (n, %) | 4 (40) | 17 (60.1) | 22 (62.9) | 0.419^c^; 0.862^d^ |
| BMI (kg/m^2^) | 22.6 ± 2.1 | 31.8 ± 4.4 | 30.1 ± 4.1 | <0.001^a^; 0.147^b^ |
| Hemoglobin A1c (%) | 5.6 ± 1.2 | 8.4 ± 1.6 | 9.3 ± 1.3 | <0.001^a^; 0.023^b^ |
| Hemoglobin (g/dL) | 14.1 ± 0.4 | 13.3 ± 1.0 | 12.7 ± 1.0 | 0.001^a^; 0.038^b^ |
| eGFR (mL/min/1.73m^2^) | 97.0 ± 2.3 | 66.8 ± 10.1 | 55.8 ± 7.7 | <0.001^a^; <0.001^b^ |
| BUN (mg/dL) | 11.2 ± 1.0 | 16.4 ± 2.1 | 18.0 ± 2.6 | <0.001^a^; 0.008^b^ |
| Serum creatinine (mg/dL) | 0.8 ± 0.2 | 0.9 ± 0.2 | 1.1 ± 0.2 | <0.001^a^; 0.023^b^ |
| Serum albumin (g/dL) | 4.4 ± 0.3 | 4.2 ± 0.6 | 3.9 ± 0.4 | 0.002^a^; 0.011^b^ |
| Albuminuria (mg/day) | 2.4 ± 0.6 | 93.9 ± 27.5 | 115.1 ± 45.2 | <0.001^a^; 0.027^b^ |

DN, diabetic nephropathy; BMI, body mass index; BUN, blood urea nitrogen; eGFR, estimated glomerular filtration rate. a, for differences among three groups using a Kruskal–Wallis test; b, for differences between WTAP low and high in DN groups using Mann–Whitney test. c, for differences among three groups and d, for differences between WTAP low and high in DN groups, using Chi square test.

**
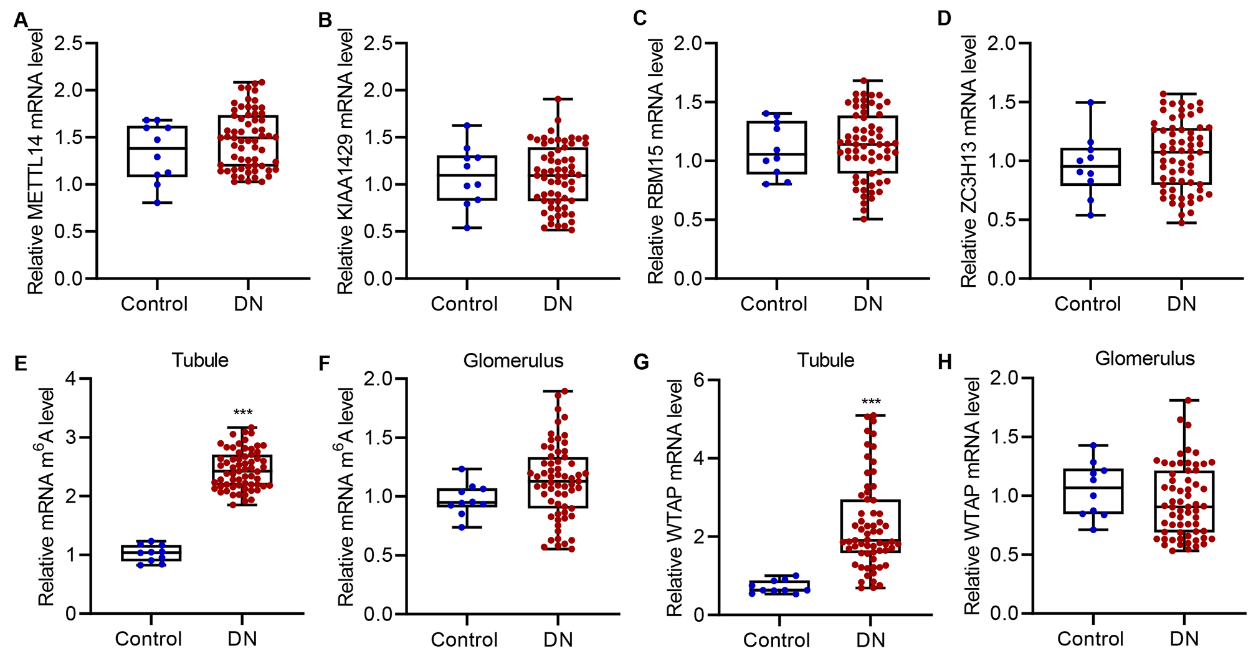
 Figure S1.** **Expression of methyltransferases** **in DN tissues.** (A–D) The mRNA expressions of METTL14, KIAA1429, RBM15 and ZC3H13 in the control (n = 10) and DN tissues (n = 63) measured by RT-qPCR. (E–F) The m^6^A levels in renal tubules and glomerulus of patinets with DN (n = 63) and control (n = 10) measured by ELISA. (G–H) The mRNA expressions of WTAP in renal tubules and glomerulus of patinets with DN (n = 63) and control (n = 10) measured by RT-qPCR. An unpaired Student's t test was used for the analysis between two groups. ****P* < 0.001 compared with control.


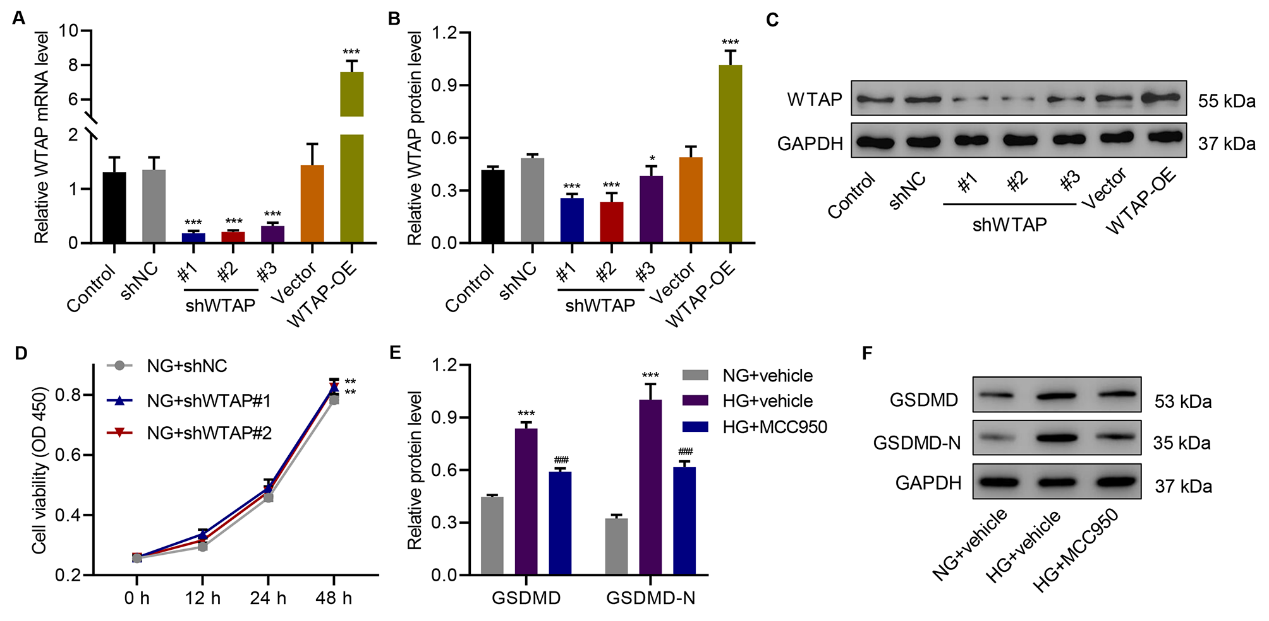


**Figure S2. WTAP, GSDMD and GSDMD-N expression and cell viability in HK-2 cells.** (A-C) Relative mRNA and protein expression of WTAP in HK-2 cells transduced with WTAP shRNA vector or overexpression vector was detected by RT-qPCR and Western blot (n = 3). (D) Cell viability in HK-2 cells treated with NG with or without WTAP knockdown (n = 3). (E, F) GSDMD and GSDMD-N expression in HK-2 cells treated with NG or HG with or without 1 μM MCC950 (n = 3). One-way analysis of variance was used to analyze the data among multiple groups, followed by Tukey's post hoc test. **P* < 0.05, ***P* < 0.01, ****P* < 0.001 compared with shNC, vector, NG+shNC or NG+vehicle. ^###^*P* < 0.001 compared with HG+vehicle.

**
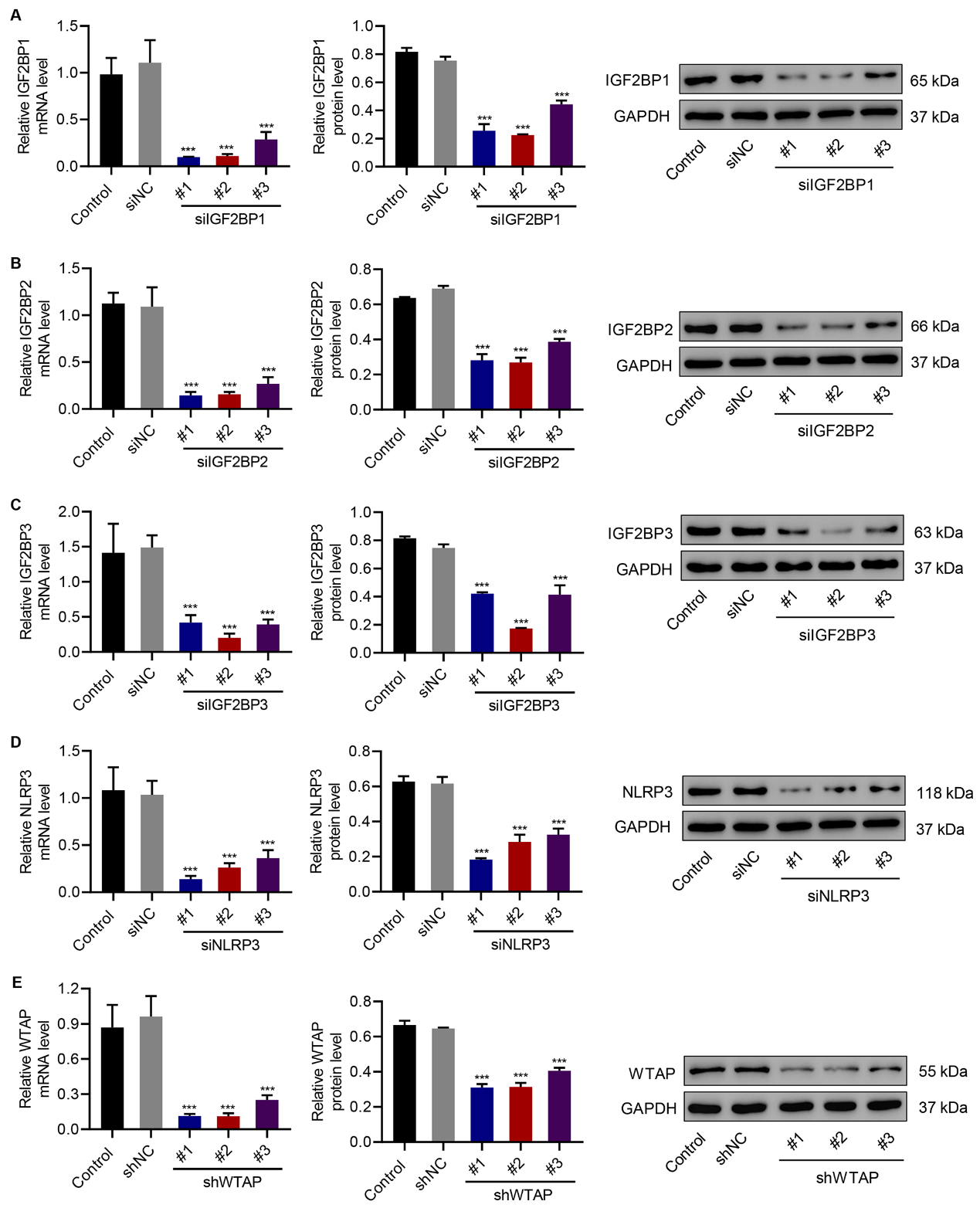
**

**Figure S3. IGF2BP and NLRP3 knockdown in HK-2 and TCMK-1 cells.** Relative mRNA and protein expression of (A) IGF2BP1, (B) IGF2BP2, (C) IGF2BP3, and (D) NLRP3 in HK-2 cells transfected with IGF2BP1, IGF2BP2, IGF2BP3, and NLRP3 siRNA vector was determined by RT-qPCR and Western blot (n=3). (E) Relative mRNA and protein expression of WTAP in TCMK-1 cells transduced with WTAP shRNA vector was detected by RT-qPCR and Western blot (n = 3). One-way analysis of variance was used to analyze the data among multiple groups, followed by Tukey's post hoc test. ****P* < 0.001 in comparison with siNC or shNC.
